# Reconstruction of parietal bone defects with adiposederived mesenchymal stem cells. Experimental study^1^

**DOI:** 10.1590/ACB351201

**Published:** 2021-01-20

**Authors:** Diego Dias da Silva, Ana Helena da Rosa Paz, Ciro Paz Portinho, Elizabeth Obino Cirne Lima, Lúcia Maria Kliemann, Marcus Vinicius Martins Collares

**Affiliations:** I MSc, Postgraduate Program in Surgical Sciences, Universidade Federal do Rio Grande do Sul, Porto Alegre-RS, Brazil.; II PhD, Assistant Professor, Department of Morphological Sciences, Universidade Federal do Rio Grande do Sul, Porto Alegre-RS, Brazil.; III PhD, Assistant Professor of Surgery, Universidade Federal do Rio Grande do Sul, Porto Alegre-RS, Brazil.; IV PhD, Associate Professor, School of Veterinary Medicine, Universidade Federal do Rio Grande do Sul, Porto Alegre-RS, Brazil.; V PhD, Assistant Professor of Pathology, Hospital de Clínicas de Porto Alegre, Universidade Federal do Rio Grande do Sul, Porto Alegre-RS, Brazil.; VI PhD, Associate Professor of Surgery, Universidade Federal do Rio Grande do Sul, Porto Alegre-RS, Brazil.

**Keywords:** Bone Regeneration, Platelet-Rich Plasma, Tissue Engineering, Rats

## Abstract

**Purpose:**

This study assessed the regeneration potential of mesenchymal stem cells (MSC) from adipose tissue associated with platelet-rich plasma (PRP) in bone regeneration.

**Methods:**

Thirty Wistar rats (Rattus norvegicus albinos) were divided into five groups (according to the grafting material and time to euthanasia): (1) autograft - 14 days (control), (2) autograft - 28 days (control), (3) MSC + PRP - 14 days, (4) MSC + PRP + papaverine - 14 days and (5) MSC + PRP + papaverine - 28 days. After euthanasia, the graft was removed and histological slides were prepared. They were assessed by a blinded pathologist using a previously published histological scale as parameter.

**Results:**

There was some degree of neoformed bone trabeculae (NBT) in 93.3% of the samples, as well as osteoblastic activity (OA). The autograft groups (14 and 28 days) had higher levels in the formation of bone trabeculae. Nonparametric data were analyzed using the Wilcoxon-Mann-Whitney test and proved not to be statistically significant at p < 0.05.

**Conclusions:**

Experimental parietal bone reconstruction, combining MSC, PRP and papaverine presented regeneration in all groups with no significant difference among them.

## Introduction

Tissue engineering aims to develop new sources or ways of providing tissue for the reconstruction of destructed or damaged body areas^1^^,^ 2. In the craniofacial reconstructions there can be difficulties in obtaining bone grafts. It occurs mainly when there are extensive areas of bone loss or deformity, or when it is a case of multiple interventions^3^.

Although many synthetic substitutes have been produced, cell grafts remain the best choice, with greater capacity of integration and regeneration^3^^,^ 4. Several works on tissue engineering show that, in order to obtain the ideal means for bone regeneration, the graft must have cells with osteogenic potential, osteogenic growth factors and a matrix that serves as a mechanic mold (scaffold*)* to facilitate cellular revascularization and the tissue architecture^3^–^6^.

Even though bone marrow stem cells are the object of many studies and research, its clinical use requires elaborate procedures, which refer patients to procedures with a certain degree of morbidity due to their locations. Besides, the availability of the tissue for removal is scarce^3^^,^ 7.

With the discovery in the early 2000’s of a new source of adipose-derived mesenchymal stem cells (ADSC), new clinical perspectives have been presented due to the greater availability of tissue for removal, since fat is easily located. Moreover, the removal process has lower morbidity and is already routine in plastic surgery, which leads to a greater acceptance among patients^8^–^10^. Studies in animal models demonstrate osteogenesis capacity of the ADSC^3^^,^ 11. Lendeckel *et al.* 12 reported the use of ADSC and fibrin glue as coadjuvants to grafts in the treatment of a calvary defect.

The platelet-rich plasma (PRP) is an autologous platelet concentrate that presents growth factors and proteins with osteoconductive properties, which acts on epithelial migration and on bone and connective tissues formation^13^^,^ 14. It is obtained after blood processing, via differential centrifugation, which enables blood cell separation^15^–^17^. Platelet-rich plasma gel is achieved by addition of thrombin and calcium gluconate, which activates the coagulation system, thus generating a gelatinous product that facilitates its surgical application, also enabling platelet activation^18^^,^ 19. Platelets act in hemostasis, wound healing and reepithelization. Besides, the presence of growth factors, released by them, acts on angiogenesis, promoting vascular growth, fibroblasts proliferation and a consequent increase in collagen synthesis^13^^,^ 20.

Platelet-rich plasma has been studied in medicine and odontology and employed in bone graft surgeries in the alveolar area, in implantology, and periodontal and maxillofacial surgeries^13^^,^ 21. In a clinical study with a sample of 20 patients who underwent dental extraction before the insertion of implants, Anitua reported that the alveoli treated with PRP presented greater buccolingual thickness at the moment of inserting the implants. Reepithelization was also better when compared with the group that did not receive PRP^16^.

The studies in medicine demonstrate great potential of PRP in improving the results of orthopedic and neurosurgical procedures, as well as of those in plastic surgery^20^^,^ 22–24. In rhytidoplasty, abdominoplasty and mammoplasty surgeries and when there is the presence of skin flaps, PRP helps in hemostasis and stimulates neovascularization, thus decreasing complications, such as hematomas, seromas and flap suffering^22^^,^ 24. This study aims to assess the potential of the association of ADSC (extracted from the gonadal fat of rats, isolated, cultivated and expanded in laboratory) with a PRP scaffold and papaverine (a vasodilator drug) as a bone substitute.

## Methods

This is an open, compared, prospective experimental study conducted in the Universidade Federal do Rio Grande do Sul (UFRGS) and Hospital de Clínicas de Porto Alegre (HCPA). The participating units were the Unit of Craniomaxillofacial Surgery of the Division of Plastic Surgery of HCPA, the Laboratory of Embryology and Cellular Differentiation of HCPA, the Unit of Animal Experimentation (UEA) at the Center of Experimental Research (CPE) of HCPA and the Department of Pathology of the Medical School of UFRGS and the Unit of Experimental Pathology Unit of HCPA.

All procedures were reviewed and approved by HCPA Ethics Committee, which follows the rules for animal experimentation, advised by the Council for International Organization of Medical Sciences (CIOMS). This study is in accordance to the Animal Research: Reporting of In Vivo Experiments (ARRIVE) guidelines statement.

Thirty adult male Wistar rats with an average of 74 days-old and average weight of 302.24 g were included. The study groups were divided as follows:

- Group 1: Autograft and euthanasia in 14 days (SG 14);
- Group 2: Autograft and euthanasia in 28 days (SG 28);
- Group 3: ADSC graft + PRP and euthanasia in 14 days (ADSC + PRP 14);
- Group 4: ADSC graft + PRP + papaverine and euthanasia in 14 days (ADSC + PRP + PPV 14);
- Group 5: ADSC graft + PRP + papaverine and euthanasia in 28 days (ADSC + PRP + PPV 28).

### Experimental model of bone defect

Anesthesia was administered to the Wistar rats with ketamine [100 mg/kg intraperitoneal; Vetbrands, Jacareí (SP), Brazil] and xylazine [10 mg/kg subperitoneal; Vetbrands, Jacareí (SP), Brazil]. Local anesthetic was bupivacaine 0.5% (1 mg/kg). After that, trichotomy was conducted in the area, leaving the head exposed. Iodophor aqueous solution was applied over the animal’s head for antiseptics, isolating the operative region with sterile fields.

An incision was made in the shape of a scythe in order to isolate the graft area from contact with the incised and sutured area. Then, the flap was lifted and the skullcap periosteum was scraped with a scalpel blade in the site of the future defect. Next, the size of the defect was marked with a sterile pen.

Total thickness bone defects were created (with a flexible, double-face, cutting, diamond disc; KG Sorensen, Brazil) in the region of the parietal bone, to be reconstructed in the same surgical time, according to each study group. After that, a delicate periosteal dissector was employed to open the defect, minimizing meningeal laceration.

After the removal of the portion of the skull cap, the graft was added, according to the group in question, and the scalp was closed with a simple suture stitch (4-0 or 5-0 nylon). Next, the animals were placed in appropriate incubator for surgical recovery, at 37 °C.

In the postoperative period, the animals were kept in cages with cycles of 12 h of light, with access to water and food *ad libitum*. Analgesia was conducted with tramadol [1 mg/kg, intramuscular, 8/8 h, two doses; Carlo Erba SA., Duque de Caxias (RJ), Brazil].

The animals were euthanized at the postoperative either 14 or 28 days’ time point in a CO_2_ camera [Biotécnicas, São Paulo (SP), Brazil], according to the routine at the Unit of Animal Experimentation at the CPE, HCPA. We decided on those periods because it has been demonstrated that graft osteogenesis starts from two to four weeks^25^.

### Isolation of mesenchymal stem cells

Two adult Wistar rats weighing approximately 300 g were used as tissue donors for cell isolation. The gonadal fat was collected and then processed in the Laboratory of Embryology and Cellular Differentiation (CPE at the HCPA).

After the euthanasia of the animals, the adipose tissue was removed under sterile conditions and processed in a laminar flow cabinet. Next, the tissue was put in collagenase solution type I [0.5 mg/mL in Dulbecco’s modified Eagle’s medium (DMEM) 10 mM HEPES] for a period of 45 minutes at 37 °C to promote tissue digestion. After the complete digestion, the enzyme was inactivated by the addition of DMEM supplemented with 10% of fetal bovine serum (FBS).

After isolation, the cells were cultivated in DMEM, containing low concentration of glucose (Invitrogen, CA, USA), supplemented with 15 mM Hepes, 15% fetal bovine (Invitrogen, CA, USA) and antibiotic solution of 100 units/mLof penicillin and 100 mg/mL of streptomycin (Gibco, NM, USA) at 37 °C in atmosphere of 5% CO_2_ and 100% humidity. After 24 h of cultivation, the culture medium was aspirated and half a flask was added. When the cell culture presented a confluence of 80%, the adherent cells were removed with a solution of 0.05% trypsin-EDTA (Gibco, NM, USA) for posterior subculture in DMEM with 10% FBS (complete medium).

After the second passage of the cells, they could already be grafted. The cells were preserved, frozen and 5 days before the surgery were defrosted and prepared for grafting.

### Characterization of the culture of mesenchymal stem cells

Based on the consensus published by the International Society for Cellular Therapy, the ADSC used in this work was characterized according to morphology, immunophenotyping and differentiation *in vitro.*

The immunophenotypic analysis consisted of a panel of antibodies for positive and negative selection. The expressions of CD90, CD29 and CD34 were tested. The antibodies were used in 1:100 dilution. The analyses were conducted on the flow cytometer BD FACSCalibur (Becton & Dickinson, NJ, USA) from the Department of Biochemistry at UFRGS and the results were analyzed via software Paint-A-Gate.

The *in vitro* differentiation was performed. Three different experiments were conducted for the ADSC differentiation induction: osteogenic, adipogenic and chondrogenic differentiation. For the osteogenic differentiation, the DMEM 15 mM Hepes induction medium was used, supplemented with 10^-8^ mol/L of dexamethasone (Sigma, MO, USA), 5 μg/μL of Ascorbic acid 2-phosphate (Sigma, MO, USA) and 10 mM/L of β-Glycerophosphate (Sigma MO, USA) in ADSC culture up to 21 days. Osteogenic differentiation was detected by alizarin red staining [Nuclear, Sao Paulo (SP), Brazil], which stains the extracellular matrix rich in calcium. For the adipogenic differentiation, the ADSC was cultivated in DMEM 15 mM Hepes, 10^-8^ mol/L of dexamethasone (Sigma, MO, USA), 5 μg/mL of insulin and 50 μg/mL of indomethacin (Sigma, MO, USA). The adipogenic differentiation was detected 21 days after the beginning of the differentiation test via staining with Oil Red (Sigma, MO, USA), which stains the fat vacuole. In the chondrogenic differentiation, a DMEM 15 mM medium was used, supplemented with Hepes 6.25 ug/mL, insulin 10 ng/mL, transforming growth factor (TGF) beta1 and 50 nM ascorbic acid 2-phosphate. The detection of differentiation was conducted through staining with *Alcian Blue*, which has affinity for the anionic groups present in the glycosaminoglycans of the extracellular matrix.

### Preparation of platelet-rich plasma– protocol of Sonnleitner

The blood collection for the production of the PRP gel was done in the immediate preoperative (after the anesthesia) according to the Sonnleitner protocol^26^. With the use of a microhematocrit, 1 mL of blood was collected from the retro-orbital plexus directly in an Eppendorf flask containing sodium citrate to prevent coagulation. Next, it was briefly agitated and put in a centrifuge for 20 min at 760 RPM (160 G). At the end of the process, three components were obtained inside the tube, separated in layers. The red blood cells on the bottom, the PRP in the middle and the platelet-poor plasma (PPP) on top. The middle and superior parts of the tube were removed with a pipette (penetrating lightly the red blood cell layer in order to effectively collect the PRP) for a new Eppendorf and centrifuged again.

Centrifugation was repeated for 15 minutes at 1200 RPM (400 G). Previously, it had been 20 minutes at 760 RPM (160 G). After that, calcium gluconate was added and, after 10 more minutes, the consistency of gel was obtained, which is characteristic of fibrin glue.

### Transplant of the cells

After the conclusion of the bone defect and the PRP gel preparation, the cells contained in an Eppendorf tube were removed with a pipette and added to the tube with the PRP gel. The cellular concentration in each sample was approximately 5 × 10^5^ cells/mL. Then the graft was placed on the defect and, according to the group, papaverine (0.05 mL/sample) was added.

*Histological analysis*

After the euthanasia, the grafted areas were removed to enable the histological analysis. The removed grafted areas were put in formalin and sent to the Unit of Experimental Pathology for histological preparation and posterior staining with hematoxylin-eosin (HE). The removed graft was dried and fixated in formalin at 10%. Next, the material was descaled in nitric acid solution at 10% for a minimum period of 24 h. The material was cut longitudinally in slices and totally underwent the routine histological processing. From each block 4-micra thick cuts were stained in HE and then 30 slides were produced.

The histological analysis was conducted using the modified histological scale of Portinho^27^ (Table 1). From the original scale, the parameters of neoformed bone trabeculae (NBT) and osteoblastic activity (OA) were analyzed.

**Table 1:** Histological scale.

Criterium		Score		Score description
Neoformed bone trabeculae (NBT)		0		Absence of neoformed trabeculae
		1		Thin, isolated trabeculae, not surpassing 1/3 of the microscope field
		2		Isolated or anastomosing trabeculae, occupying 1/3 to 2/3 of the microscope field.
		3		Thick trabeculae, predominantly anastomosing, occupying more than 2/3 of the microscope field
Osteoblastic activity (OA)		0		Nonexistent activity
		1		Less than 1/3 of the neoformed trabeculae presents OA
		2		Activity observed in 1/3 to 2/3 of the NBT
		3		More than 2/3 of the neoformed trabeculae present OA

Modified from Portinho^27^.

### Statistical analysis

The groups were analyzed with the Wilcoxon–Mann–Whitney test, for the NBT and OA, since the variables presented a nonparametric distribution.

### Sample size estimation

The sample size estimation was performed in PEPI version 4.0 and based on Portinho’s study^27^. For a significance level of 5%, a variance of 10 with a 5% margin of error, a minimum total of 30 animals was estimated.

## Results

**Figure 1:**
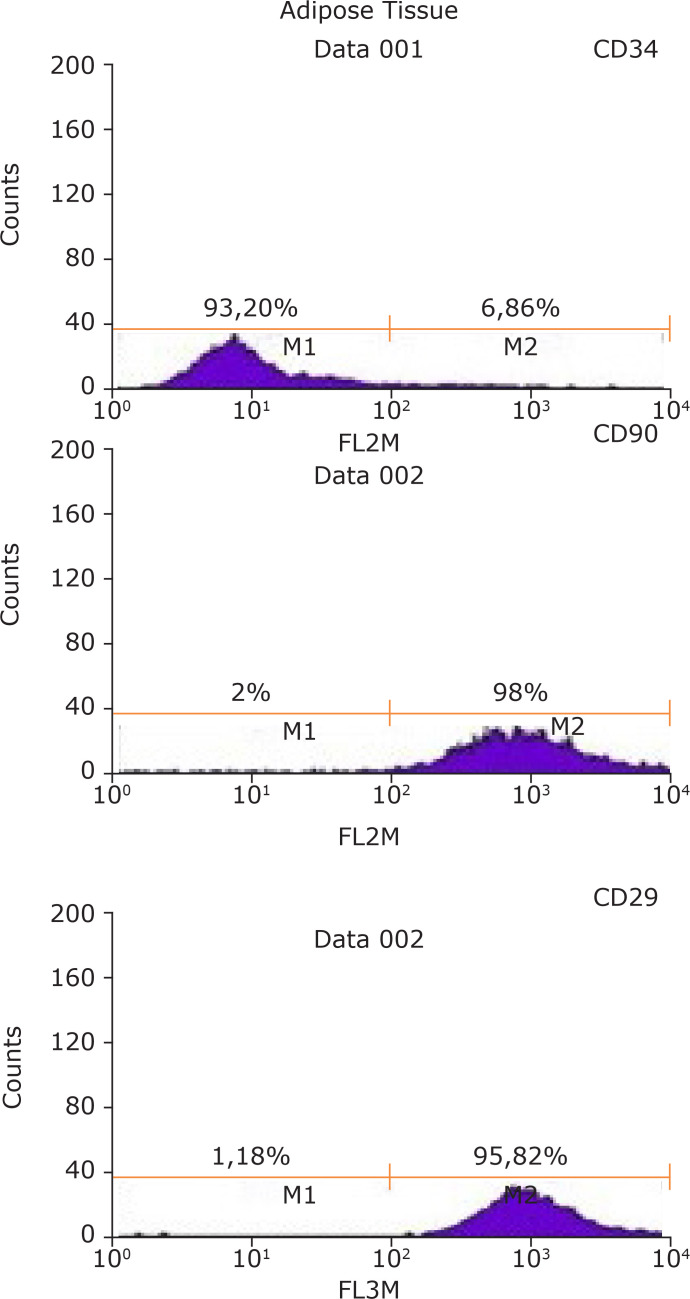
Immunophenotypic profile of MSCs derived from different sources. Flow cytometry histograms show the expression of selected molecules (CD34, CD90 and CD29).

The immunophenotyping profile of adipose tissue derived stem cells used in the study is depicted in Fig. 1. Flow cytometry histograms revealed that 93.20% are negative for CD34. Also, 98% of the cells are positive for CD90 and 95.82% are positive for CD29. This is consistent with the immunophenotyping profile of adipose tissue derived stem cells.

**Figure 2:**
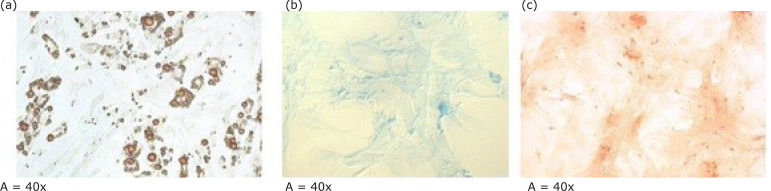
Differentiation of MSCs from adipose tissue. Mesenchymal stem cells were cultured in adipogenic, chondrogenic and osteogenic medium. Lipid vacuoles are stained orange with Oil Red O.(a)Sulfated proteoglycans deposits are stained blue with Alcian Blue.(b)Calcium deposited in the extracellular matrix is stained red by Alizarin Red. (c) Magnifications and cell lines are indicated below each image.

Mesenchymal stem cells (MST) differentiation from adipose tissue is illustrated in Fig. 2. The MST were cultivated in adipogenic, chondrogenic and osteogenic media.

Groups 1, 3 and 4 (euthanasia in 14 days) were compared, as well as groups 2 and 5 (euthanasia in 28 days). Some degree of NBT was obtained in 93.3% of the total sample, as well as OA (score 1, 2 or 3 on Portinho^27^ modified histological scale). The 6.7% that did not present NBT nor OA belonged to group 5 (Tables 2 and 3).

**Table 2:** Frequency of neoformed bone trabeculae.

Histological score		n		%
0		2		6.7
≥ 1		28		93.3
Total		30		100

0 = no trabecula formed; ≥ 1 = there was trabecular formation.

**Table 3:** Frequency of osteoblastic activity.

Score		n		%
0		2		6.7
≥ 1		28		93.3
Total		30		100

0 = no activity; ≥ 1 = activity present.

Groups 1 and 2 (Autograft 14 and 28 days, respectively) presented better levels of NBT than group 3 (ADSC + PRP 14 days), 4 (ADSC + PRP + PPV 14 days) and 5 (ADSC + PRP + PPV 28 days) (Table 4). Osteoblastic activity was higher in groups 1 and 4 in comparison to groups 2, 3 and 5 (Table 5). However, the differences observed in the NBT and OA parameters were not statistically significant (p < 0.05).

**Table 4:** Median of the score for the histological criterium of NBT, by group.

Group		Description		Median
1		AG 14		2
2		AG 28		2
3		PRP + SC 14		1
4		PRP + SC + PPV 14		1
5		PRP + SC + PPV 28		1

AG: autograft; SC: stem cells; PPV: papaverine; PRP: platelet rich plasm.

**Table 5:** Median of the score for the histological criterium of OA, by group.

Group		Group		Median
1		AG 14		3
2		AG 28		2
3		PRP + SC 14		2
4		PRP + SC + PPV 14		3
5		PRP + SC + PPV 28		2

AG: autograft; SC: stem cells; PPV: papaverine; PRP: platelet rich plasm.

The difference between autograft and cellular groups did not prove to be statistically significant for the parameters of bone trabeculae (G1 × G3, p = 0.097; G1 × G4, p = 0.530; G2 × G5, p = 0.268). Considering the OA, no significant difference was found among the groups (G1 × G3, p = 0.620; G1 × G4, p = 0.876; G2 × G5, p = 0.639).

A significant difference was not found between the groups with or without papaverine in relation to the formation of bone trabeculae (G3 × G5, p = 0.755). Concerning OA, no statistically significant difference was detected either (G3 × G5, p = 1.000).

## Discussion

The majority of the prior studies utilized mesenchymal bone marrow-derived stem cells (BMSC). Adipose-derived mesenchymal stem cells were used due to the advantage of the adipose tissue being abundant and easy to obtain. Besides, obtaining BMSC is more difficult than ADSC and the procedure is more morbid for the patient^8^^,^ 9. Several authors showed that the ADSC could differentiate into pluripotential cells and produce bone tissue *in vitro* and *in vivo* 3 ^,^ 8 ^,^ 9 ^,^ 28 ^,^ 29.

There was formation of bone trabeculae in 93.3% of the total study sample, with a distribution of that phenomenon through all the analyzed groups. In 6.7% of the sample (2 slides), there was no bone formation (score 0 on the modified histological scale of Portinho^27^); both belonged to group 5 (ADSC + PRP + PPV 28 days).

In the groups involving stem cells (60% of the samples, 18 out of 30 animals), we obtained (in 88.9%, 16 of 18 samples) some degree of formation of bone trabeculae, which demonstrates the osteogenic potential of the ADSC already reported in the literature^3^^,^ 12 ^,^ 27 ^,^ 30. In the group involving autograft, the formation of bone trabeculae was 100%. That is in accordance with the literature, where autograft, for its properties of osteoconduction, osteoinduction and osteogenesis, remain the gold standard^27^^,^ 31.

The autograft groups (groups 1 and 2) performed better in the NBT when compared with the groups with cells (groups 3, 4 and 5), but there was no statistical difference^32^–^36^. That might have occurred in part for the small size of each group (six animals per group), although the calculus of the sample allowed that population. Also, the PRP scaffold may not be the best cell carrier, albeit that is still to be studied.

As for the OA parameter, as well as the NBT, the autograft group presented 100% of activity to 88.23% of the groups with cells and the samples that did not present OA belonged to group 5.

Groups 1 (AG 14 days) and 4 (ADSC + PRP + PPV 14 days) obtained better scores in comparison to groups 2, 3 and 5. Osteoblastic activity can be an important element since, in spite of the low neoformation of bone trabeculae in the groups with cells (3, 4 and 5), those trabeculae presented an OA equivalent to the autograft 28 days, in groups 3 and 5 and equivalent to group AE 14 days in group 4.

Since the OA is an indicator of bone cellular activity in the region, it is expected that, with a longer observation time, it is possible to reach similar levels of bone formation among the groups. The results demonstrating a superiority of autograft in the parameters analyzed (NBT and OA) also corroborate the literature^32^–^37^.

Platelet-rich plasma was used as scaffold due to the fact that it is an autogenous element, easy to obtain and one that has in its composition inflammatory response mediator factors and adjuvants in the wound healing process (PDGF, TGF-β, IGFs), besides its adhesive capacity. However, the materials used as scaffold in the literature have the common characteristic of the presence of micropores in their structure, which benefits and generates a stimulus to the graft cells. That may have been a factor, besides the use of undifferentiated ADSC, through which the grafts did not have a superior result in relation to autograft.

Di Bella *et al*.^30^ reported that large pores are necessary to guarantee vascular growth and tissue formation. A highly interconnected network of micropores is necessary to allow cell-cell communication. Moreover, pores between 250 and 400 μm prove to be cellular adhesion and tissue formation conductive.

It may be necessary to assess the PRP not as the main scaffold, but as adjuvant, for its growth factors, to other materials [lyophilized bone, poly lactic glycolic acid, bone morphogenetic protein (BPM)]. According to Tobita^38^, the effectiveness of the PRP in the bone regeneration is not clear, particularly because the PRP does not present BMP, which is a more potent osteoinductive protein that promotes the differentiation of stem cells for the osteoblastic lineage and that can induce the bone formation, including the ectopic.

Papaverine is a potent vasodilator and has been used to improve circulation in expanded skin flaps. Thus, its vasodilating action could improve vascular bed, facilitating the integration of the graft and decreasing the hypoxia caused in the tissues by the surgical act. In experimental works, its topic use decreased the function of the myofibroblasts and improved circulation^39^. Papaverine also increases the blood flow of microvascular anastomosis^40^^,^ 41. However, no significant effect was found among the groups with or without the addition of papaverine, either in 14 or 28 days. There was bone regeneration in all the groups but without statistical difference with the addition of the studied components (ADSC, papaverine and PRP), nor between the studied periods (14 versus 28 days). Further research with longer follow up periods is necessary to clarify this issue.

## Conclusion

Experimental parietal bone reconstruction, combining MSC, PRP, and papaverine presented regeneration in all the groups with no significant difference among them.
